# Publisher Correction: Accurate estimation of cell composition in bulk expression through robust integration of single-cell information

**DOI:** 10.1038/s41467-020-16607-9

**Published:** 2020-06-03

**Authors:** Brandon Jew, Marcus Alvarez, Elior Rahmani, Zong Miao, Arthur Ko, Kristina M. Garske, Jae Hoon Sul, Kirsi H. Pietiläinen, Päivi Pajukanta, Eran Halperin

**Affiliations:** 1 Bioinformatics Interdepartmental Program, UCLA, Los Angeles, CA 90095 USA; 2 Department of Human Genetics, David Geffen School ofMedicine at UCLA, Los Angeles, CA 90095 USA; 3 Department of Computer Science, School of Engineering, UCLA, Los Angeles, CA 90095 USA; 4 Department of Psychiatry and Biobehavioral Sciences, UCLA, Los Angeles, CA 90095 USA; 5 Obesity Research Unit, Research Program for Clinical and Molecular Metabolism, University of Helsinki, Helsinki, 00014 Finland; 6 Obesity Center, Endocrinology Abdominal Center, Helsinki University Central Hospital and University of Helsinki, Helsinki, 00260 Finland; 7 Institute for Precision Health, School of Medicine, UCLA, Los Angeles, CA 90095 USA; 8 Department of Anesthesiology, UCLA Health, Los Angeles, CA 90095 USA; 9 Department of Computational Medicine, School of Medicine, UCLA, Los Angeles, CA 90095 USA

**Keywords:** Computational biology and bioinformatics, Gene expression, RNA sequencing

Correction to: *Nature Communications* 10.1038/s41467-020-15816-6, published online 24 April 2020.

The original version of this article contained an error in the author affiliations. The affiliation of Eran Halperin with Institute for Precision Health, School of Medicine, UCLA, Los Angeles, CA 90095, USA was inadvertently omitted. This has now been corrected in both the PDF and HTML versions of the article.

