# Supplementary material for: Accurate estimation of cell composition in bulk expression through robust integration of single-cell information
Source: Nat Commun. 2020 Apr 24;11:1971. doi: 10.1038/s41467-020-15816-6 (PMC7181686; doi:10.1038/s41467-020-15816-6)
Supplement: Supplementary file 1 — Supplementary Information [file 41467_2020_15816_MOESM1_ESM.pdf]

Supplementary Information for:

Jew et al. “Accurate estimation of cell composition in bulk expression through robust integration of single-cell information”

Supplementary Tables

a Association of adipocyte proportion estimates in adipose tissue with BMI

**Method**	**Spearman Correlation**	**Spearman p-value**	**Effect Estimate**	**Effect Standard Error**	**Effect t-value**	**Effect p-value**
Bisque	-0.178	0.090	-0.282	0.126	-2.240	**0.030**
MuSiC	0.038	0.719	-0.081	0.108	-0.754	0.455
BSEQ-sc	-	-	-	-	-	-
CIBERSORT	-	-	-	-	-	-
CIBERSORTx	-0.300	**0.004**	-0.361	0.100	-3.624	**0.001**
BisqueMarker	-0.227	**0.030**	-0.304	0.096	-3.154	**0.003**

b Association of macrophage proportion estimates in adipose tissue with BMI

**Method**	**Spearman Correlation**	**Spearman p-value**	**Effect Estimate**	**Effect Standard Error**	**Effect t-value**	**Effect p-value**
Bisque	0.389	**1.291e-04**	0.460	0.099	4.671	**3.078e-05**
MuSiC	0.065	0.540	0.034	0.110	0.308	0.760
BSEQ-sc	0.238	**0.022**	0.278	0.092	3.013	**0.004**
CIBERSORT	0.239	**0.022**	0.162	0.102	1.597	0.118
CIBERSORTx	0.273	**0.009**	0.224	0.102	2.192	**0.034**
BisqueMarker	0.296	**0.004**	0.253	0.103	2.465	**0.018**

c Association of T cell proportion estimates in adipose tissue with Matsuda index

**Method**	**Spearman Correlation**	**Spearman p-value**	**Effect Estimate**	**Effect Standard Error**	**Effect t-value**	**Effect p-value**
Bisque	-0.195	0.075	-0.387	0.116	-3.328	**0.002**
MuSiC	-	-	-	-	-	-
BSEQ-sc	-	-	-	-	-	-
CIBERSORT	-	-	-	-	-	-
CIBERSORTx	-0.317	**0.003**	-0.230	0.111	-2.068	**0.046**
BisqueMarker	-0.294	**0.007**	-0.188	0.100	-1.874	0.069

Supplementary Table 1: Significance of associations of estimated cell proportions and measured phenotypes in 100 subcutaneous adipose tissue samples. We fit a linear mixed-effects model (LMM) to account for the twin structure of the dataset as a random effect, with additional fixed effects to account for age, age-squared, and sex. Expected effect directions were based on previously reported findings. An entry of '-' indicates that the method did not detect the indicated cell population in any of the samples. Bold values were found to be significant at $\alpha = 0.05$ and in expected directions.
a Association of adipocyte proportion with BMI. A negative association was expected.
b Association of macrophage proportion with BMI. A positive association was expected.
c Association of T cell proportion with Matusda index, a measure of insulin resistance. A negative association was expected. An additional covariate accounting for diabetes status was added to the LMM due to previously reported significant associations with Matsuda index.
Source data are provided as a Source Data file.

a Association of neuron proportion estimates in DLPFC tissue with cognitive diagnosis

Method	Spearman Correlation	Spearman p-value	Effect Estimate	Effect Standard Error	Effect t-value	Effect p-value
Bisque	-0.167	**3.123e-05**	-0.145	0.039	-3.705	**2.305e-04**
MuSiC	-0.167	**3.102e-05**	-0.147	0.039	-3.742	**1.995e-04**
BSEQ-sc	-0.142	**3.944e-04**	-0.053	0.039	-1.341	0.180
CIBERSORT	-0.173	**1.643e-05**	-0.155	0.039	-3.971	**7.998e-05**
CIBERSORTx	-0.162	**5.229e-05**	-0.127	0.039	-3.237	**0.001**
BisqueMarker	-0.141	**4.383e-04**	-0.142	0.039	-3.645	**2.897e-04**

b Association of microglia proportion estimates in DLPFC tissue with Braak stage

Method	Spearman Correlation	Spearman p-value	Effect Estimate	Effect Standard Error	Effect t-value	Effect p-value
Bisque	0.094	**0.018**	0.118	0.037	3.220	**0.001**
MuSiC	0.057	0.151	0.019	0.037	0.509	0.611
BSEQ-sc	-0.190	1.683e-06	-0.166	0.037	-4.525	7.244e-06
CIBERSORT	0.003	0.943	-0.005	0.037	-0.137	0.891
CIBERSORTx	0.109	**0.006**	0.056	0.037	1.517	0.130
BisqueMarker	0.092	**0.021**	0.054	0.037	1.444	0.149

Supplementary Table 2: Significance of associations of estimated cell proportions and measured phenotypes in 628 DLPFC tissue samples. We fit a linear model with covariates to account for age, age-squared, and sex. Expected effect directions were based on previously reported findings. Bold values were found to be significant at $\alpha = 0.05$ and in expected directions.
a Association of neuron proportion with cognitive diagnosis category. A negative association was expected.
b Association of microglia proportion with Braak stage, a measure of neurofibrillary tangles. A positive association was expected.
Source data are provided as a Source Data file.

Supplementary Figures

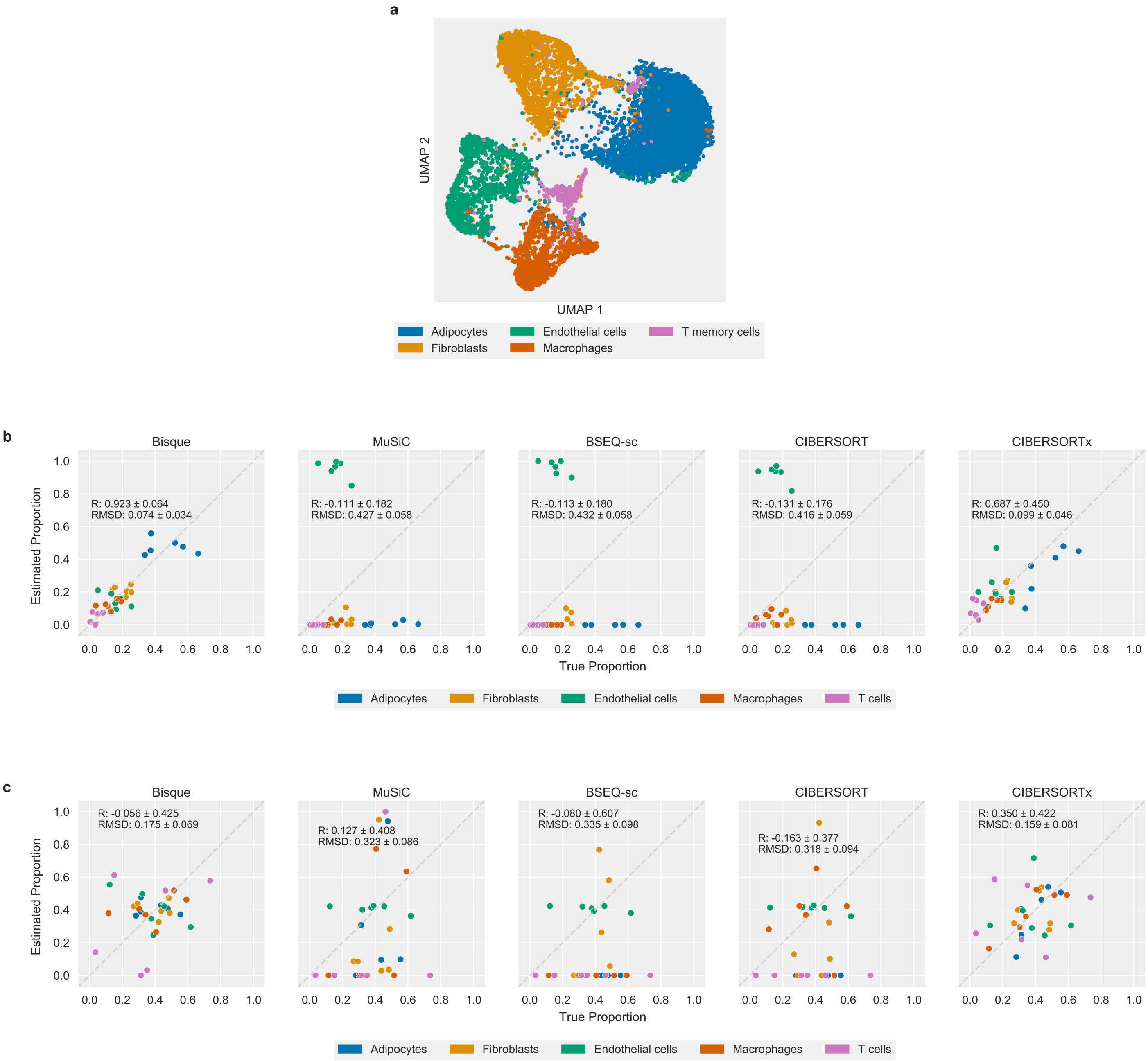

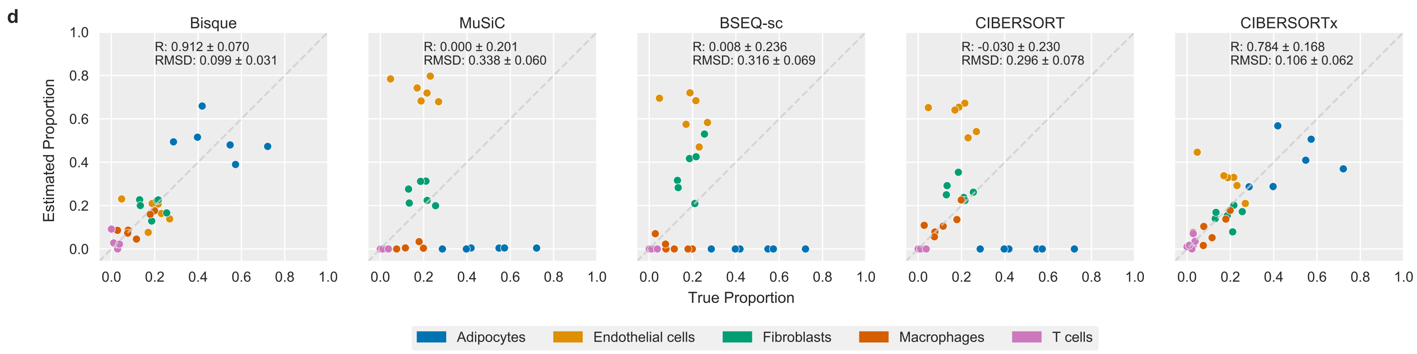

Supplementary Figure 1: Decomposition of human subcutaneous adipose tissue.
a UMAP projection of snRNA-seq data with 5 identified cell type clusters labeled.
b Leave-one-out cross-validation using 6 samples with snRNA-seq and bulk RNA-seq data available. Proportions based on snRNA-seq were used as a proxy for the true proportions on the x-axis. Estimated proportions for an individual were generated by each decomposition method using the remaining 5 individuals as training data. Each color represents one of the 5 identified cell populations.
c Leave-one-out cross-validation performance after normalization of estimates within each cell type to determine cell-specific accuracy. Normalized estimates are robust to inflation of global Pearson correlation by large cell populations; however, these metrics are noisy when considering only six individuals.
d Leave-one-out cross-validation performance on exon-aligned snRNA-seq data. Existing methods are able to detect additional cell populations using the exonic subset of the snRNA-seq data, though around 40% of the sequenced cells are filtered out.
Source data are provided as a Source Data file.

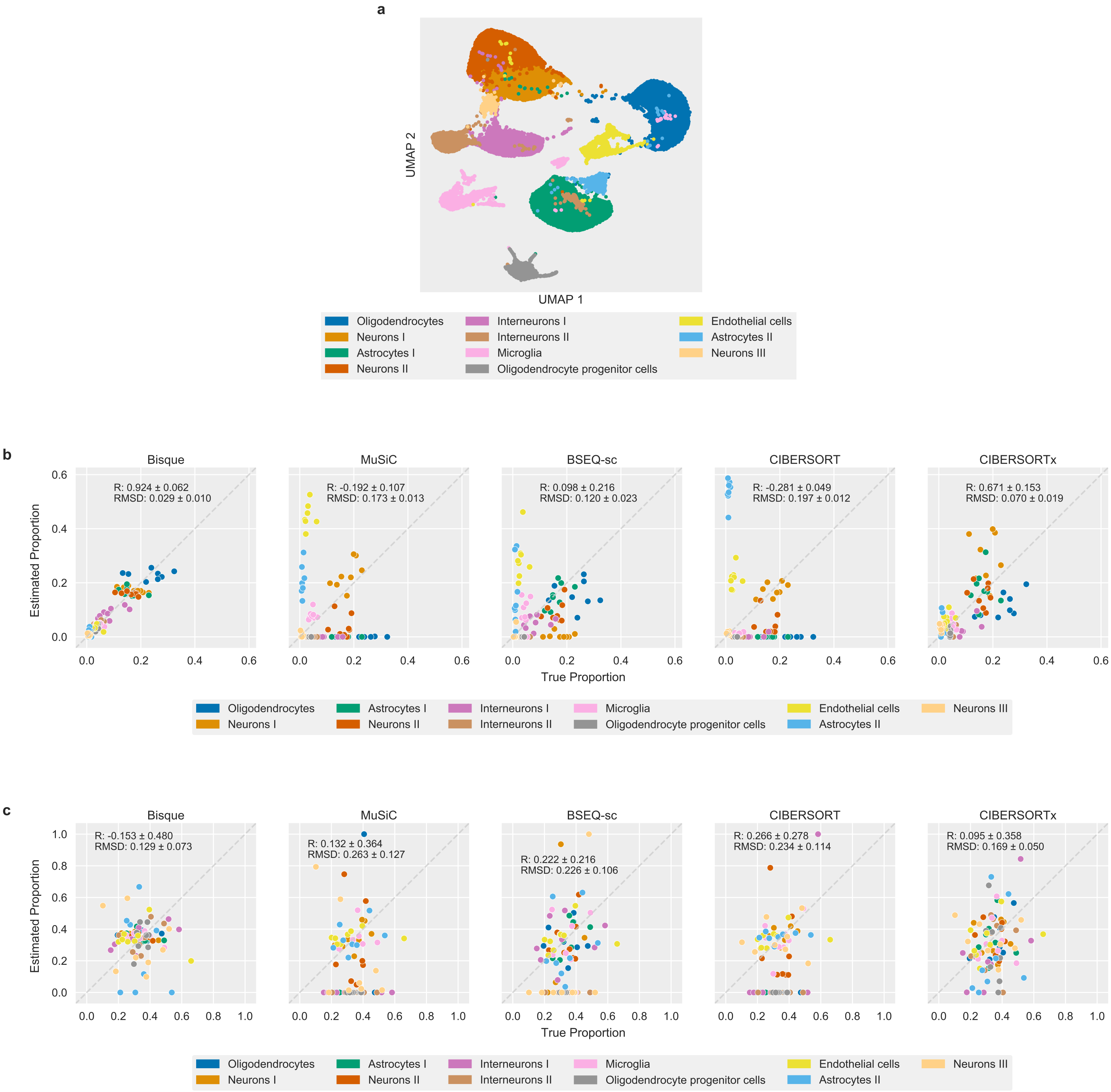
a
UMAP 2
UMAP 1
Oligodendrocytes
Neurons I
Astrocytes I
Neurons II
Interneurons I
Interneurons II
Microglia
Oligodendrocyte progenitor cells
Endothelial cells
Astrocytes II
Neurons III
b
Bisque
R: 0.924 ± 0.062
RMSD: 0.029 ± 0.010
MuSiC
R: -0.192 ± 0.107
RMSD: 0.173 ± 0.013
BSEQ-sc
R: 0.098 ± 0.216
RMSD: 0.120 ± 0.023
CIBERSORT
R: -0.281 ± 0.049
RMSD: 0.197 ± 0.012
CIBERSORTx
R: 0.671 ± 0.153
RMSD: 0.070 ± 0.019
Estimated Proportion
True Proportion
c
Bisque
R: -0.153 ± 0.480
RMSD: 0.129 ± 0.073
MuSiC
R: 0.132 ± 0.364
RMSD: 0.263 ± 0.127
BSEQ-sc
R: 0.222 ± 0.216
RMSD: 0.226 ± 0.106
CIBERSORT
R: 0.266 ± 0.278
RMSD: 0.234 ± 0.114
CIBERSORTx
R: 0.095 ± 0.358
RMSD: 0.169 ± 0.050
Estimated Proportion
True Proportion

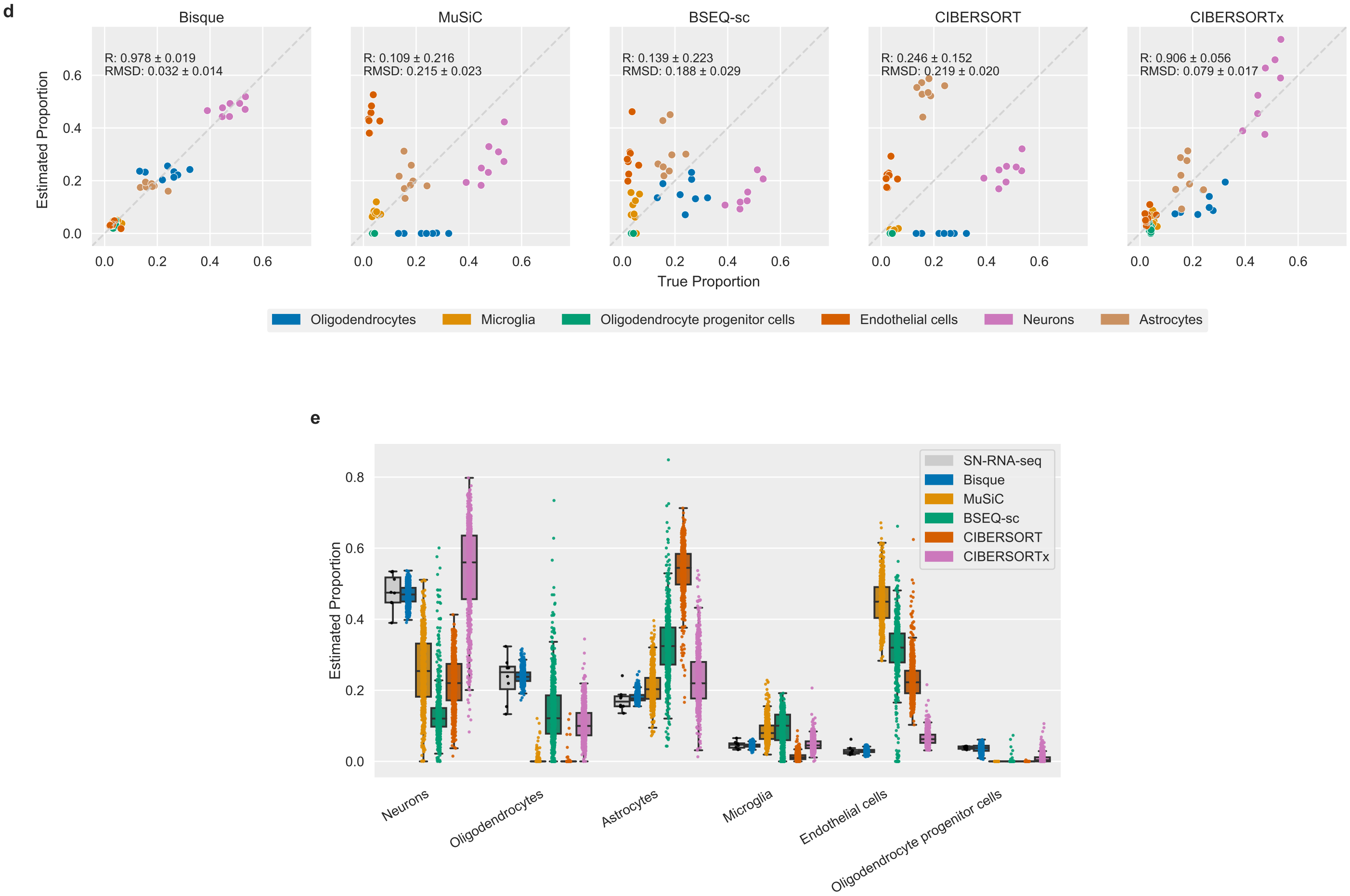

Supplementary Figure 2: Decomposition of human DLPFC tissue.
a UMAP projection of snRNA-seq data with 11 identified cell type clusters labeled.
b Leave-one-out cross validation using 8 samples with snRNA-seq and bulk RNA-seq data available. Proportions based on snRNA-seq were used as a proxy for the true proportions on the x-axis. Estimated proportions for an individual were generated by each decomposition method using the remaining 7 individuals as training data. Each color represents one of the 11 identified cell populations.
c Leave-one-out cross-validation performance after normalization of estimates within each cell type to determine cell-specific accuracy. As described previously, performance metrics on normalized data provide better measure of global accuracy but are noisy with small sample sizes.
d Leave-one-out cross-validation performance after merging closely related cell subtypes into 6 clusters. Performance of existing methods increases compared to decomposition into 11 clusters with related subtypes.
e Decomposition of remaining 628 individuals with cell subtype merging. The aggregated cell type proportions estimated from the 8 snRNA-seq samples are similar to IHC estimates for neurons and astrocytes from 70 individuals in the cohort (data not shown). Boxes indicate the quartiles of the estimated proportions with whiskers extending 1.5 times the interquartile range. Points are individual samples that are represented by the boxplot.
Source data are provided as a Source Data file.

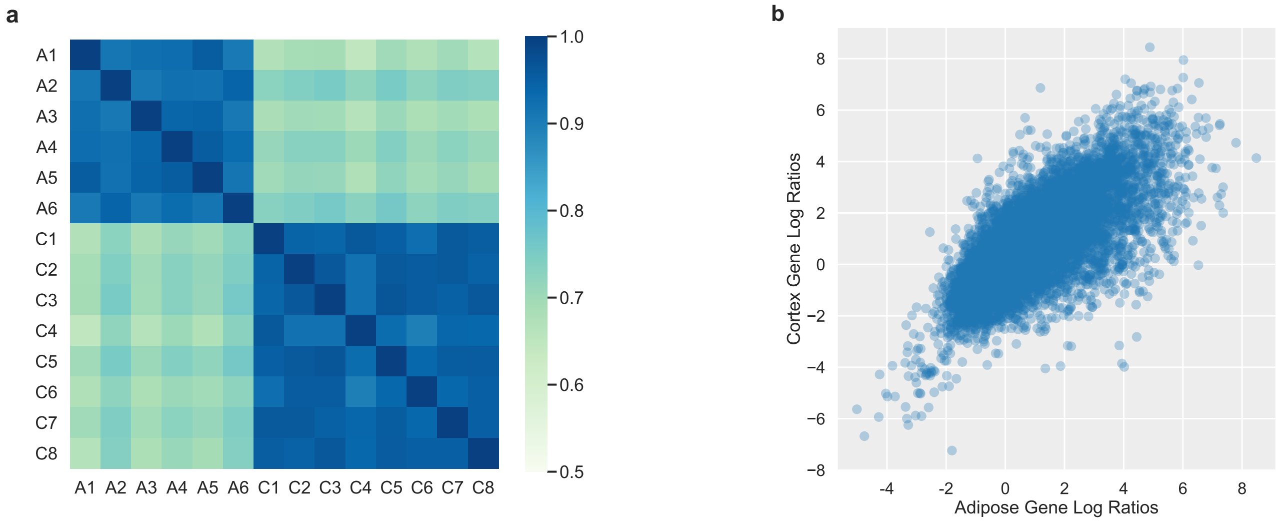

Supplementary Figure 3: Consistency of snRNA-seq to bulk RNA-seq expression log-ratios across individuals, tissues, and experiments.
a Heatmap depicting Pearson correlation between pairs of individual's log-ratios of snRNA-seq expression to bulk RNA-seq gene expression measured in counts per million (CPM). A sample prefix of 'A' indicates an individual from the adipose dataset and 'C' indicates an individual from the cortex dataset. Correlation is high between individuals within experiments as well as between experiments/tissues, indicating the same genes are over/under-expressed in snRNA-seq when compared to bulk RNA-seq.
b Scatterplot of average snRNA-seq to bulk RNA-seq gene expression log-ratios across individuals in adipose dataset (x-axis) and cortex dataset (y-axis). Each point corresponds to a gene detected in both experiments, depicting the average ratio across all individuals for that tissue. The snRNA-seq to bulk RNA-seq ratios vary across genes and correlate (R=0.747) between these two experiments.
Source data are provided as a Source Data file.

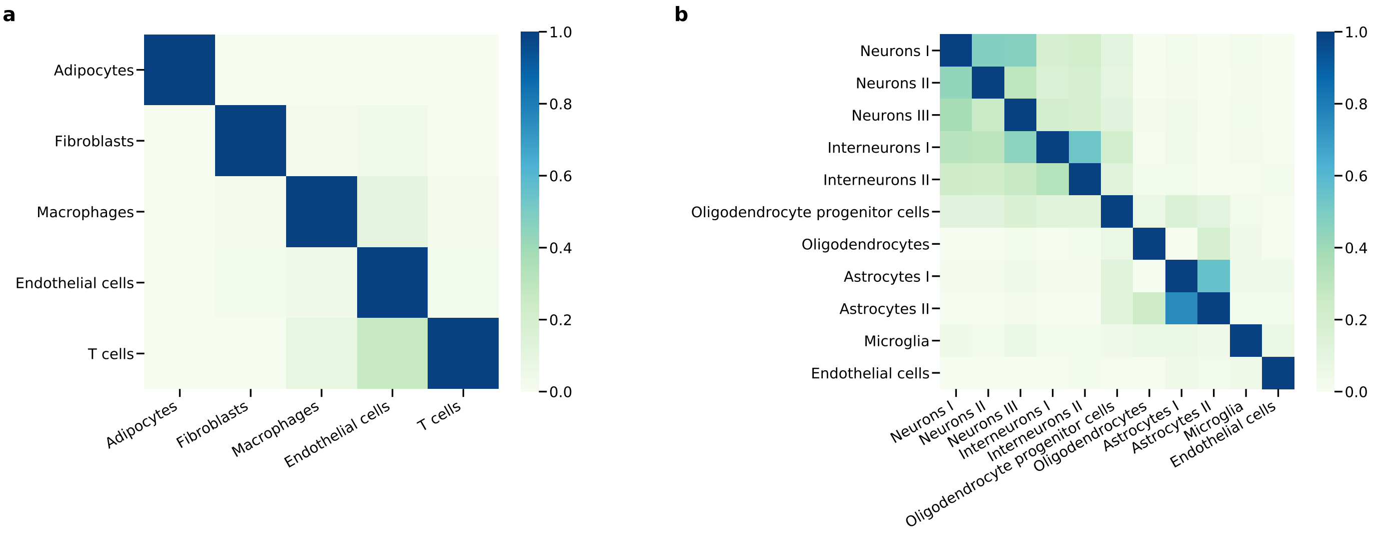

Supplementary Figure 4: Shared marker genes between identified clusters in snRNA-seq data. Heatmaps of proportion of shared marker genes where an entry indicates the proportion of marker genes for the cluster on the x-axis that are found in the cluster on the y-axis.
a The 5 clusters identified in adipose tissue are relatively distinct in their marker genes.
b The 11 clusters identified in DLPFC tissue have several closely related subtypes, such as neurons and astrocytes.
Source data are provided as a Source Data file.

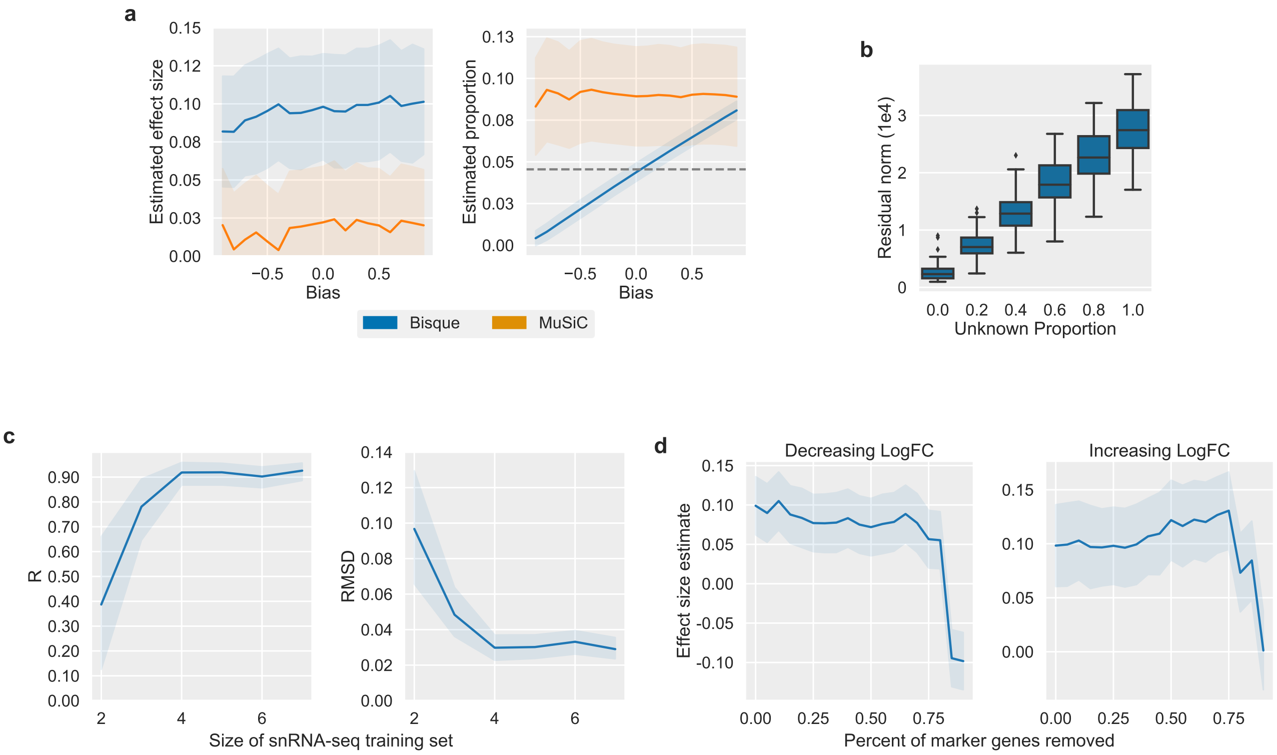

Supplementary Figure 5: Robustness of the reference-based decomposition model.
a Microglia cells in the 8 DLPFC snRNA-seq samples were upsampled or downsampled at various percentages, denoted as bias on the x-axis, to simulate reference data that may overrepresent or underrepresent a cell type of interest compared to bulk data. Decomposition performance, measured as the estimated effect size of microglia proportion on Braak stage (which is expected to be positive) on the y-axis was consistent for each method as the bias in the snRNA-seq reference varied (left). Effect sizes were fit in a linear regression model on 628 samples adjusting for age at death, age at death squared, and sex. Since Bisque utilizes proportions observed in the reference data under the assumption that they reflect physiological compositions, the simulated bias propagates to the estimated proportions (right). Shaded regions indicate standard error of estimates. On the left, the line indicates the estimated effect size of the linear model. On the right, the line indicates the mean cell proportion estimate.
b The DLPFC bulk RNA-seq data was mixed with the adipose bulk RNA-seq data at various proportions to simulate an unknown cell population in the bulk data that is not represented in the snRNA-seq reference data. In order to model the severity of the sample discordance, we compared the amount of adipose contamination, denoted as unknown proportion on the x-axis, to the residuals from the Bisque model (y-axis). As this simulated unknown proportion increases, the residuals of Bisque also increase. Each boxplot represents a distinct random subset of 100 samples from the DLPFC dataset that were mixed with 100 randomly selected samples from the adipose data. Boxes indicate the quartiles of the observed residual norms with whiskers extending 1.5 times the interquartile range. Points are outliers beyond this range.
c Leave-one-out cross-validation performance across the 8 samples in the DLPFC dataset after utilizing random subsamples of the snRNA-seq data as a reference. Performance, in terms of Pearson correlation (left) and RMSD (right), began to drop when using less than 4 individuals in the reference dataset. Shaded regions indicate 95% confidence interval with a line indicating the mean observed value.
d An increasing number of marker genes for the microglia cells in the DLPFC dataset were removed to determine the effect on decomposition performance. At each amount of genes removed (x-axis), performance was measured as the effect size of the estimated microglia proportion on Braak stage (y-axis). Effect sizes were fit in a linear regression model on 628 samples adjusting for age at death, age at death squared, and sex. Genes were removed in order of decreasing (left) or increasing (right) log-fold-change. In both settings, performance remained relatively consistent until around 75% of the 102 identified marker genes were removed. Shaded regions indicate standard error of estimated effects with a line indicating the actual estimated effect.
Source data are provided as a Source Data file.
