# Supplementary material for: Accurate estimation of cell composition in bulk expression through robust integration of single-cell information
Source: Nat Commun. 2020 Apr 24;11:1971. doi: 10.1038/s41467-020-15816-6 (PMC7181686; doi:10.1038/s41467-020-15816-6)
Supplement: Supplementary file 3 — Reporting Summary [file 41467_2020_15816_MOESM3_ESM.pdf]

nature research

Corresponding author(s): Eran Halperin
Paivi Pajukanta

Last updated by author(s): Mar 17, 2020

Reporting Summary

Nature Research wishes to improve the reproducibility of the work that we publish. This form provides structure for consistency and transparency in reporting. For further information on Nature Research policies, see Authors & Referees and the Editorial Policy Checklist.

Statistics

For all statistical analyses, confirm that the following items are present in the figure legend, table legend, main text, or Methods section.

n/a	Confirmed	
☐	☒	The exact sample size (*n*) for each experimental group/condition, given as a discrete number and unit of measurement
☐	☒	A statement on whether measurements were taken from distinct samples or whether the same sample was measured repeatedly
☐	☒	The statistical test(s) used AND whether they are one- or two-sided<br>*Only common tests should be described solely by name; describe more complex techniques in the Methods section.*
☐	☒	A description of all covariates tested
☐	☒	A description of any assumptions or corrections, such as tests of normality and adjustment for multiple comparisons
☐	☒	A full description of the statistical parameters including central tendency (e.g. means) or other basic estimates (e.g. regression coefficient) AND variation (e.g. standard deviation) or associated estimates of uncertainty (e.g. confidence intervals)
☐	☒	For null hypothesis testing, the test statistic (e.g. *F*, *t*, *r*) with confidence intervals, effect sizes, degrees of freedom and *P* value noted<br>*Give P values as exact values whenever suitable.*
☒	☐	For Bayesian analysis, information on the choice of priors and Markov chain Monte Carlo settings
☐	☒	For hierarchical and complex designs, identification of the appropriate level for tests and full reporting of outcomes
☐	☒	Estimates of effect sizes (e.g. Cohen's *d*, Pearson's *r*), indicating how they were calculated

Our web collection on statistics for biologists contains articles on many of the points above.

Software and code

Policy information about availability of computer code

Data collection	No software was used in collecting data.
Data analysis	Single-nucleus RNA-seq data were aligned using CellRanger 3.0.2 against the GRCh38.p12 genome assembly. Bulk RNA-seq data were aligned with STAR 2.5.1 and quantified using featureCounts 1.6.3, both against the GRCh38.p12 genome assembly. R 3.5.1 was used for further processing and decomposition experiments. The Seurat v3.0.0 R package was used to filter, cluster, and identify cell type marker genes from the single-nucleus data, The sctransform 0.2.0 R package was used to normalize and scale the single-nucleus data. Bisque 1.0, xbioc 0.1.7, Biobase 2.4.2, MuSiC 0.1.1, bseqsc 1.0, CIBERSORT v1.06, CIBERTSORTx were all used for decomposition using the processed bulk and single-nucleus RNA-seq data. The R nlme 3.1-127 package was used for linear-mixed-model association. All visualizations and were generated with Python 3.7.2 using Seaborn 0.9.0, Matplotlib 3.0.3, Pandas 0.24.2, and Numpy 1.16.2, sklearn 0.20.3, and scipy 1.2.1.

For manuscripts utilizing custom algorithms or software that are central to the research but not yet described in published literature, software must be made available to editors/reviewers. We strongly encourage code deposition in a community repository (e.g. GitHub). See the Nature Research guidelines for submitting code & software for further information.

Data

Policy information about availability of data

All manuscripts must include a data availability statement. This statement should provide the following information, where applicable:

- Accession codes, unique identifiers, or web links for publicly available datasets
- A list of figures that have associated raw data
- A description of any restrictions on data availability

The adipose data used in these analyses are available from the corresponding authors upon reasonable request. The cortex data are available on Synapse (10.7303/syn3219045). Single-nucleus RNA-seq data (https://www.synapse.org/#!Synapse:syn16780177), bulk RNA-seq data (https://www.synapse.org/#!Synapse:syn3388564) and phenotypes (https://www.synapse.org/#!Synapse:syn3191087) are available under controlled use conditions set by human privacy

regulations. A data use agreement is required to access these data. The source data underlying Tables 2 and 3, Figs 2, 3, 4, and 5, Supplementary Tables 1 and 2, and Supplementary Figs 1, 2, 3, 4, 5 are provided as a Source Data file.

Field-specific reporting

Please select the one below that is the best fit for your research. If you are not sure, read the appropriate sections before making your selection.

☒ Life sciences ☐ Behavioural & social sciences ☐ Ecological, evolutionary & environmental sciences

For a reference copy of the document with all sections, see nature.com/documents/nr-reporting-summary-flat.pdf

Life sciences study design

All studies must disclose on these points even when the disclosure is negative.

Sample size	The single-nucleus brain data consists of 8 samples while the bulk brain data consists of 636 samples. The single-nucleus adipose data consists of 6 samples, while the bulk adipose consists of 106. The 106 samples are part of a BMI-discordant monozygotic twin cohort, and the 6 samples are a subset of the 106. The sample sizes of the bulk RNA-seq data sets were selected to allow for associations with complex phenotypes, while the sample sizes of the single-nucleus RNA-seq data sets were selected to allow for accurate decomposition of the bulk RNA-seq. These samples sizes are comparable to previously published data sets for single-cell RNA-seq (Segerstolpe, Palasantza et al. Cell Metabolism 2016; Wilson et al. PNAS 2019; Amamoto et al. NAR 2019) and bulk tissue RNA-seq (Raulerson et al. AJHG 2019; GTEx project; Brown et al. eLife 2014).
Data exclusions	No data were excluded.
Replication	Cell type proportion-phenotype associations were not replicated with additional outside data because of lack of publicly available samples containing both single-cell and bulk RNA-seq data. The accuracy of our method was determined by leave-one-out cross validation in our 2 independent cohorts.
Randomization	There was no randomization in this study. Age, age squared, and sex were corrected using fixed effects, while twin status in the adipose data was corrected using random effects.
Blinding	Investigators collecting and generating data were unaware of the phenotypes of the samples.

Reporting for specific materials, systems and methods

We require information from authors about some types of materials, experimental systems and methods used in many studies. Here, indicate whether each material, system or method listed is relevant to your study. If you are not sure if a list item applies to your research, read the appropriate section before selecting a response.

Materials & experimental systems

n/a	Involved in the study	
☒	☐	Antibodies
☒	☐	Eukaryotic cell lines
☒	☐	Palaeontology
☒	☐	Animals and other organisms
☐	☒	Human research participants
☒	☐	Clinical data

Methods

n/a	Involved in the study	
☒	☐	ChIP-seq
☒	☐	Flow cytometry
☒	☐	MRI-based neuroimaging

Human research participants

Policy information about studies involving human research participants

Population characteristics	Adipose: The participants are of European descent, consist of 60% females and 40% males, and are 30-70 years old. They are generally healthy with rates of diabetes and obesity similar to that of the population.<br>Brain: Samples were collected from deceased individuals. All individuals were of European ancestry except 9 of African ancestry, 1 of Native American ancestry, and 1 of Asian or Pacific Island ancestry. 408 of the individuals were female and 228 were male. Individuals were aged from 71 to 90+ at time of death. Final cognitive diagnoses ranged from no cognitive impairment for 222 of the individuals to some form of cognitive impairment for 414 individuals. 1 individual did not have a cognitive diagnosis reported.
Recruitment	Adipose: participants were recruited at the University of Helsinki as mono-zygotic twins with a discordance in BMI of at least 3. Twin status is corrected for as a random effect in a mixed model.<br>Brain: No donors were recruited, the samples were obtained from participants in the Religious Order Study.<br>The twins were recruited from a national registry a medical center, lowering the potential for selection bias. The distributions of metabolic phenotypes is close to that of the population distributions, indicating self-selection bias is unlikely. The RUSH

participants were also recruited at a medical center. These patients were further selected for healthy controls and Alzheimer's Disease cases after follow-up, diminishing the possibility of a selection bias.

Ethics oversight	Adipose: University of California, Los Angeles,USA, and University of Helsinki, Finland<br>Brain: The Religious Orders Study and Rush Memory and Aging Project were approved by an IRB of Rush University Medical Center

Note that full information on the approval of the study protocol must also be provided in the manuscript.
